# Lestaurtinib is a potent inhibitor of anaplastic thyroid cancer cell line models

**DOI:** 10.1371/journal.pone.0207152

**Published:** 2018-11-12

**Authors:** Nicole Pinto, Stephenie D. Prokopec, Frederick Vizeacoumar, Karlee Searle, Matthew Lowerison, Kara M. Ruicci, John Yoo, Kevin Fung, Danielle MacNeil, Jim C. Lacefield, Hon S. Leong, Joe S. Mymryk, John W. Barrett, Alessandro Datti, Paul C. Boutros, Anthony C. Nichols

**Affiliations:** 1 Department of Otolaryngology—Head and Neck Surgery, Western University, London, Ontario, Canada; 2 Department of Anatomy and Cell Biology, Western University, London, Ontario, Canada; 3 Ontario Institute for Cancer Research, Toronto, Ontario, Canada; 4 Cancer Research Cluster, University of Saskatchewan, Saskatchewan, Canada; 5 Western University, London, Ontario, Canada; 6 Department of Medical Biophysics, Western University, London, Ontario, Canada; 7 Department of Urology, Mayo Clinic, Rochester, Minnesota, United States of America; 8 Department of Oncology, Western University, London, Ontario, Canada; 9 Department of Microbiology and Immunology, Western University, London, Ontario, Canada; 10 Network Biology Collaborative Centre, Lunenfeld-Tanenbaum Research Institute, Mount Sinai Hospital, Toronto, Ontario, Canada; 11 Department of Agricultural, Food, and Environmental Sciences, University of Perugia, Perugia, Italy; 12 Department of Medical Biophysics, University of Toronto, Toronto, Ontario, Canada; 13 Department of Pharmacology & Toxicology, University of Toronto, Toronto, Ontario, Canada; University of South Alabama Mitchell Cancer Institute, UNITED STATES

## Abstract

Anaplastic thyroid cancer (ATC) is a rare and lethal human malignancy with no known effective therapies in the majority of cases. Despite the use of conventional treatments such as chemotherapy, radiation and surgical resection, this disease remains almost universally fatal. In the present study, we identified the JAK2 inhibitor Lestaurtinib as a potent compound when testing against 13 ATC cell lines. Lestaurtinib demonstrated a potent antiproliferative effect *in vitro* at nanomolar concentrations. Furthermore, Lestaurtinib impeded cell migration and the ability to form colonies from single cells using scratch-wound and colony formation assays, respectively. Flow cytometry was used for cell cycle analysis following drug treatment and demonstrated arrest at the G2/M phase of the cell cycle, indicative of a cytostatic effect. *In vivo* studies using the chick chorioallantoic membrane xenograft models demonstrated that treatment with Lestaurtinib resulted in a significant decrease in endpoint tumor volume and vascularity using power Doppler ultrasound imaging. Overall, this study provides evidence that Lestaurtinib is a potent antiproliferative agent with potential antiangiogenic activity that warrants further investigation as a targeted therapy for ATC.

## Introduction

Thyroid cancer is the most common endocrine malignancy[1]. Well-differentiated thyroid cancers make up the majority of thyroid cancers and have an excellent prognosis[2]. In contrast, anaplastic thyroid cancer (ATC) is a rare type of undifferentiated thyroid cancer that makes up approximately 1% of thyroid cancer cases and is arguably the most lethal human malignancy[3–5]. Patients diagnosed with ATC typically present with a rapidly expanding neck mass resulting in airway and esophageal obstruction, and distant metastases[6,7]. Despite the aggressive use of chemotherapy, radiation and surgical resection, the outcomes for patients with ATC remain dismal, with a mean survival of only 6 months[6,8]. While there have been studies to date with the aim of understanding the molecular pathogenesis of disease, it is evident that ATC is still very poorly understood[9–11].

Presently, there are no effective therapies for patients diagnosed with ATC and therefore, the use of targeted agents directed against specific genetic alterations and signaling pathways remains an attractive cancer treatment strategy. Small-molecule tyrosine kinase inhibitors represent a molecularly-precise method of cancer treatment that can be used to target specific signaling pathways and produce an antiproliferative effect[12,13]. Indeed, kinase inhibitors are undergoing active investigation in every major cancer type and have been shown to provide meaningful therapeutic responses in recurrent and metastatic diseases, with increased cure rates when administered concurrently or in the adjuvant setting with surgery or radiation[14–16]. While a small number of targeted agents have been tested in patients with ATC, there are currently no therapies that have been approved for routine treatment of ATC[17].

To begin to fill the gap in our understanding of this disease and how it can be treated, we screened 13 ATC cell lines and identified Lestaurtinib as a highly potent agent with nanomolar potency. Efficacy of Lestaurtinib was further validated both *in vitro* and *in vivo* using the chick chorioallantoic membrane (CAM) xenograft model.

## Materials and methods

### Cell lines and culture conditions

THJ-11T, -16T, -21T, and -29T were all obtained from Dr. John Copland of the Mayo Clinic. U-Hth7, U-HTh74cl.7, C643, and SW1736 cell lines were obtained from Dr. Nils Erik Heldin (University of Uppsala, Sweden). Cell lines 8505C, ASH3 and KMH2 were all purchased from the Japanese Collection of Research of Bioresources Cell Bank (JCRB). Lastly, BHT-101 and CAL62 were both purchased from the DSMZ Cell Bank.

THJ-11T, -16T, -21T, and -29T cell lines were cultured in RPMI 1640 media supplemented with 10% FBS (GIBCO), 1x non-essential amino acids (Wisent), 1 mM sodium pyruvate (Wisent), penicillin (100 μg/mL) and streptomycin (100 μg/mL) (Invitrogen). U-Hth7, U-HTh74cl.7, C643, SW1736 and 8505C cell lines were cultured in EMEM media supplemented with 10% FBS (GIBCO), penicillin (100 μg/mL) and streptomycin (100 μg/mL) (Invitrogen). ASH3 and KMH2 cell lines were cultured in a 1:1 mixture of DMEM and RPMI 1640, which was supplemented with 10% heat-inactivated FBS (GIBCO), penicillin (100 μg/mL) and streptomycin (100 μg/mL) (Invitrogen). BHT-101 and CAL62 cell lines were cultured in DMEM supplemented with 10% heat-inactivated FBS (GIBCO), 1% human serum (Wisent), penicillin (100 μg/mL) and streptomycin (100 μg/mL) (Invitrogen).

### Short tandem repeat (STR) profiling of ATC cell lines

DNA was extracted from cultured cells using the AllPrep DNA/RNA/Protein kit (Qiagen), using the instructions provided by the manufacturer. A total of 100 ng of DNA per cell line was analyzed by short tandem repeat (STR) profiling at The Center for Applied Genomics (TCAG, Toronto, Canada). Cell lines were genotyped with 16 selected markers (including the 8 Combined DNA Index System (CODIS)) core STR loci, employed by the American Type Culture Collection (ATCC) and confirmed against published information (**S1 Table**).

### Drug selection

A Beckman BioMek FX liquid handler was used to dispense cells into 384-well culture plates (Corning, NY, USA) at a density of 12,000 cells/ml in a total volume of 50 μl/well. Seeded cells were incubated for 24 hours (h) at 37°C, 5% CO_2_. Lestaurtinib was initially assembled as 1mM stocks in 100% DMSO and pinned (200 nL) into wells 24 h post-seeding. Drug testing was carried out using six different concentrations ranging between 0.16 and 4 μM (final concentrations) and applied to the 13 cell lines (2 biological replicates). Negative controls consisted of wells in the same 384-well culture plate containing cells that were treated with 0.4% DMSO vehicle alone. Wells containing media only were used as a measure to determine assay background noise. To measure the percent inhibition of growth, 5 μl/well of AlamarBlue (Invitrogen) was added to each well after a 48 h incubation period of the cells with the Lestaurtinib. After the addition of AlamarBlue, cells were incubated for 4 h at 37°C and then fluorescence intensity was measured in a PHERAstar microplate reader (BMG LABTECH) equipped with 535nm excitation and 590 nm emission filters. Drug testing was completed in duplicate.

Raw data were loaded into the R statistical environment (v3.2.1). Normalized absorbance values were obtained by contrasting experimental absorbance values with those from vehicle control (DMSO-treated) cells. The half-maximal inhibitory concentrations (IC_50_ in μM) were calculated using the nplr package (v0.1–4). Similarly, the activity area (AA) was calculated as the area under the percent (%) inhibition curve using the nplr (v0.1–4) package for R. Results were filtered to remove those with poorly fitted models (R-squared < 0.9). Cell lines were classified as resistant to a compound if absorbance increased or remained constant over time; likewise, classification as sensitive required an IC_50_ within the range examined (0.16–4 μM) with the 85% confidence interval ± 2 μM. Consensus clustering using the ConsensusClusterPlus.custom (v1.8.1) package for R was used to find groups of cell lines with similar response patterns (based on activity area).

### Reagents

The tyrosine kinase inhibitor Lestaurtinib (CEP-701) was purchased from Tocris (Cephalon Inc.). Lestaurtinib was dissolved in dimethyl sulfoxide (DMSO) as a 10-mM stock. Subsequent serial dilutions were performed for specific concentrations as shown in the Results section.

### Growth curves

Cells were seeded at a density of 2,400 cells/well into a 96-well plate and incubated overnight. After 24 h, cells were treated with 0.5 and 4.0 μM concentrations of Lestaurtinib. Cells were incubated with PrestoBlue (Thermo Fisher Scientific) for 1 h at 37°C and plates were read at 0, 24, 48 and 72 h after the addition of the drug. Fluorescence readings were completed using a BioTek Synergy Microplate Reader with 560nm excitation and 590nm emission wavelengths. Three technical replicates were used for each concentration tested. A Student’s unpaired, two-tail *t*-test was used for statistical analysis using Prism 7 Graphpad Software, where comparisons were made between treated and untreated samples for each timepoint. *P* values < 0.05 were considered to represent statistically significant differences. Statistical analysis is displayed for the 72 h timepoint when compared to the control at the same timepoint.

### Dose-response curves

Cells (KMH2, CAL62 and THJ-21T) were seeded at a density of 2,400 cells/well into a 96-well plate and incubated overnight. After a 24 h incubation period, cells were treated with increasing concentrations of Lestaurtinib (0.06 to 4.0 μM) for 72 h. After 72 h, cells were incubated with PrestoBlue for 1 h at 37°C. The same procedure was completed using the normal cell line, WI-38, with increasing concentrations of Lestaurtinib (0.125 to 2.0 μM) for 72 h. For ATC cell lines, two biological replicates were completed with 3 technical replicates for each concentration. For WI-38, one biological replicate was completed with 3 technical replicates for each concentration. Fluorescence readings were completed using a BioTek Synergy Microplate Reader with 560nm excitation and 590nm emission wavelengths. These values were normalized to the untreated controls and the average viability for each concentration was calculated. In order to determine the half-maximal inhibitory concentration (IC_50_), the normalized relative fluorescence values of the Lestaurtinib-treated samples were calculated as a percentage of the mean relative fluorescence units of the untreated, control samples. Drug concentrations were transformed to a logarithmic scale and IC_50_ values were calculated using non-linear regression (curve fit). Analysis was completed using Prism 7 Graphpad Software.

### Immunoblotting

Cells were seeded at a density of 200,000 cells/well into 6-well plates and incubated overnight. For treatments, cells were treated with DMSO-only (vehicle) or Lestaurtinib concentrations of 0.125, 0.25, 0.5, 1.0, 2.0 and 4.0 μM for 24 h. Staurosporine-treated cells (5 μM, 3 h) were used as a positive control for apoptosis. Cells were lysed, and a Bradford assay was performed to determine the protein concentration of the whole cell lysates using the Bio-rad Protein Assay Dye Reagent (Bio-Rad). NuPAGE Novex 4–12% Bis-Tris Gel (ThermoFisher Scientific) was used for protein separation with 20 μg of total protein loaded per well and run at 200V (1 hour). Novex Sharp Standard protein ladder was used to determine the mass of products. A PVDF blotting membrane (GE Healthcare) was used for protein transfer at 14V (1 hour). Membranes were blocked using 5% bovine serum albumin (Sigma-Aldrich). Primary antibodies were prepared according to the manufacturer and used overnight at 4°C with constant, mild agitation. Endogenous JAK2 (Cat no. 3230P), phospho-JAK2 (p-JAK2; Cat no. 3776S), phospho-STAT5 (p-STAT5; Cat no. 9351S) and α-tubulin (Cat no. 2125S) antibodies were obtained from Cell Signaling Technology. Endogenous STAT5 (Cat no. sc-835) was obtained from Santa Cruz Biotechnology. Mouse anti-human PARP (Cat no. 556494) was obtained from BD Pharminogen. Secondary antibodies were diluted 1:5000. Detection of target proteins was performed using Luminata Forte Western HRP substrate (EMD Millipore). Detection of α-tubulin was used as a loading control.

### Colony formation assay

Cells (CAL62) were seeded into 24-well plates at a density of 1,000 cells/well. After 24 h, cells were treated with 0, .125, .25, .50, 1.0, 2.0 and 4.0 μM concentrations of Lestaurtinib, with 1 biological replicate, and incubated at 37°C for 7 days with Lestaurtinib-containing media being replaced every 3 days. Cells were washed with PBS and fixed with cold 100% methanol for 15 minutes prior to staining with 0.5% crystal violet in 10% methanol/1x PBS for 10 minutes. Brightfield microscopy was used for quantification of colonies, which were defined as being ≥50 cells. A total of 3 representative fields per well were counted for colonies based on the above cell number parameter. This was completed for each concentration of Lestaurtinib tested and the number of colonies were totaled for each concentration. A Student’s unpaired, two-tail *t*-test was used for statistical analysis using Prism 7 Graphpad Software. Samples were compared relative to the untreated control and *p* values < 0.05 were considered to represent statistically significant differences in colony formation.

### Cell motility assay

Cells (CAL62) were seeded into 6-well plates at a density of 150,000 cells/well and incubated until 90% confluence. After 24 h, cells were washed with PBS and scratched through the middle in a straight line using a sterile P1000 pipette tip. Immediately after the scratch, wells were either exposed to media containing the vehicle (DMSO) or media containing Lestaurtinib at 0.5- or 4.0 μM. The migration of cells from the outer edge of the scratch towards the center was measured at 0, 4 and 24 h. A total of 10 measurements over the length of the scratch were taken for each timepoint to produce the mean scratch width (pixels), for 3 replicates per time point. Means and standard errors were calculated and normalized to the 0 h timepoint. A Student’s unpaired, two-tail *t*-test was used for statistical analysis using Prism 7 Graphpad Software and *p* values < 0.05 were considered statistically significant.

### JAK2 knockout by CRISPR/Cas9

Two cell lines (KMH2 and CAL62) were used for CRISPR/Cas9 gene editing of *JAK2*. Cells of each line were seeded at a density of 5 x 10^4^ cells/well in a 24-well dish. Lentivirus expressing Cas9 was added to cells at a multiplicity of infection (MOI) of 0.3. When integration had occurred, blasticidin selection was used to kill untransduced cells, with a concentration that was previously determined with kill curves for each cell line. *Cas9* expression was confirmed by Western blot analysis. The Cas9 (+) cell lines were then transduced with lentivirus carrying a single guide RNA (sgRNA) against *JAK2* (NM_004972, targets Exon19; Cat no. VSGH10142-246580412; Dharmacon), a scrambled non-targeting control (negative control), or a sgRNA against *DNMT3B* (positive control). Transduced cells were selected using puromycin (using a concentration determined by kill curves for each cell line) and confirmed using western blot analysis probing for endogenous *JAK2*. Proliferation was compared between the non-targeting CRISPR control and *JAK2* knockout lines where cell lines were seeded in 96-well plates at a density of 2,400 cells/well. After 72 h, PrestoBlue was added to wells and plates were incubated for 1 h prior to fluorescence readings using a BioTek Synergy H4 Hybrid Microplate Reader with 560nm excitation and 590nm emission wavelengths. Relative fluorescence units were normalized to yield percent (%) proliferation compared to the non-targeting control. Furthermore, we completed dose-response curves where CAL62 and KMH2:JAK2 knockout (KO) cell lines were seeded into 96-well plates at a density of 2,400 cells/well. At 24 h post-seeding, Lestaurtinib was added at a range of increasing concentrations (0.125–4 μM). After 72 h, PrestoBlue was added to wells and plates were read as mentioned above. A Student’s unpaired, two-tail *t*-test was used for statistical analysis using Prism 7 Graphpad Software and *p* values < 0.05 were considered statistically significant differences.

### Live-dead cell assay

Cells (KMH2 and CAL62) were seeded at a density of 100,000 cells/well. After 24 h, cells were either untreated or treated with 0.5 μM or 1 μM Lestaurtinib. Plates were incubated at 37°C for 24 h. Cells were then washed with PBS and trypsinized; 10 μl of resuspended cells were combined with Trypan Blue (Sigma-Aldrich; Cat. T8154) at a 1:1 ratio for 10 minutes. Cells were counted using a hemocytometer and Brightfield microscopy. The ratio of live-dead cells was then calculated for each based on their exclusion of dye. The number of live cells per mL were calculated and compared to the untreated control using a Student’s unpaired, two-tail *t*-test. *P* values < 0.05 were considered to be statistically significant.

### Cell-cycle analysis

To determine the effects of Lestaurtinib on cell cycle distribution, we treated two ATC cell lines (CAL62 and KMH2) with either DMSO-only or 4 μM Lestaurtinib for 24 h, with 3 biological replicates each. This concentration was chosen from the upper range of the high-throughput drug screen, and both cell lines were picked due to their sensitivity as noted from the IC_50_ analysis. Bromo-deoxyuridine (BrdU; GE Healthcare, Cat. RPN201) was added to cell media and incubated in culture for 2 h prior to collection. Cell were trypsinized, collected and washed 3 times with PBS prior to using 95% freezer-chilled ethanol to fix cells. Cells were permeabilized using 2N HCl/0.5% Triton X-100, followed by 0.1 M NaB_4_O_7_. Primary (Mouse anti-BrdU primary antibody, 1:50; BD Biosciences lot. 347580) and secondary (FITC-conjugated rabbit anti-mouse secondary antibody, 1:25; Vector Laboratories Cat. FI-2000) antibodies were added and incubated for 30 minutes for each step. Cell pellets were resuspended in propidium iodide (PI; 10 mg/ml) and RNase A (0.25 mg/ml; Bioshop Canada Inc., Cat. RNA675) and incubated overnight at 4°C. The following day, cell cycle analysis using flow cytometry was performed using a Beckman Coulter Inc, Cytomics FC500 flow cytometer with at least 10,000 events that were counted per test. Statistical analysis was performed using a Student’s unpaired, two-tail *t*-test to compare treated and untreated samples.

### Antitumor activity studies *in vivo*

One million cells of the CAL62 cell line were combined with Matrigel matrix at a 1:1 ratio. Chick embryos were used at 9 days post-fertilization. A small circle of filter paper was placed on an area with small blood vessels of the chorioallantoic membrane to score the area for the onplant of cells. A total volume of 10 μl, containing the 1:1 ratio of 5 μl 1 million cells: 5 μl Matrigel, was pipetted over top of the scored area. Once the onplant of cells was complete, embryos were covered and incubated for 2 days at 37°C. Drug treatments were initiated 2 days post-onplant with either vehicle (DMSO) (n = 11) or 4 μM Lestaurtinib (n = 19) and applied once daily for a 5-day duration. Tumor volume was measured at the experimental endpoint using B-mode (anatomical) ultrasound imaging. Vascularity was measured at endpoint using power Doppler ultrasound imaging, which estimates fractional vascular volume by displaying the ultrasound power reflected from moving blood cells in detectable vessels, which are typically larger than 100 μm diameter.

All ultrasound measurements were performed using a Vevo 2100 high frequency imaging system (VisualSonics Inc.). B-mode and power Doppler images were acquired using a 40 MHz linear array (MS-550D, 40 μm axial resolution, 80 μm lateral resolution). The imaging field of view was set to 10.00 mm (axial) by and 14.08 mm (lateral) with a Doppler color box sized to completely enclose the largest cross-section of tumor. Three-dimensional volumetric images were acquired using a linear stepper motor (VisualSonics Inc.) with a step size 0.076 mm and a total step distance 7.98 mm.

Volumetric image reconstruction and analysis was performed using the supplied software (Vevo 2100 Workstation, VisualSonics Inc.). Estimates of tumor volume were computed *via* manual planimetry on reconstructed three-dimensional B-mode images. The fractional vascular blood volume in the tumor was calculated by summing the number of Doppler color voxels, which correspond to regions with detected blood flow, within the segmented volume and dividing by the total number of voxels in the volume to yield percent vascularity. This yields the percentage tumor vascularity, which is normalized to the tumor volume.

### Statistical analysis

A Student’s unpaired, two-tail *t*-test was performed where appropriate using Prism 7 Graphpad Software Macintosh Version (by Software MacKiev 1994–2014 GraphPad Software, Inc). *P* values < 0.05 were considered statistically significant. *P* values are defined as ns *p* > 0.05, * represents *p* < 0.05, ** represents *p* < 0.01, and *** represents *p* < 0.001.

## Results

### Lestaurtinib inhibits growth of ATC cell lines

**Fig 1:**
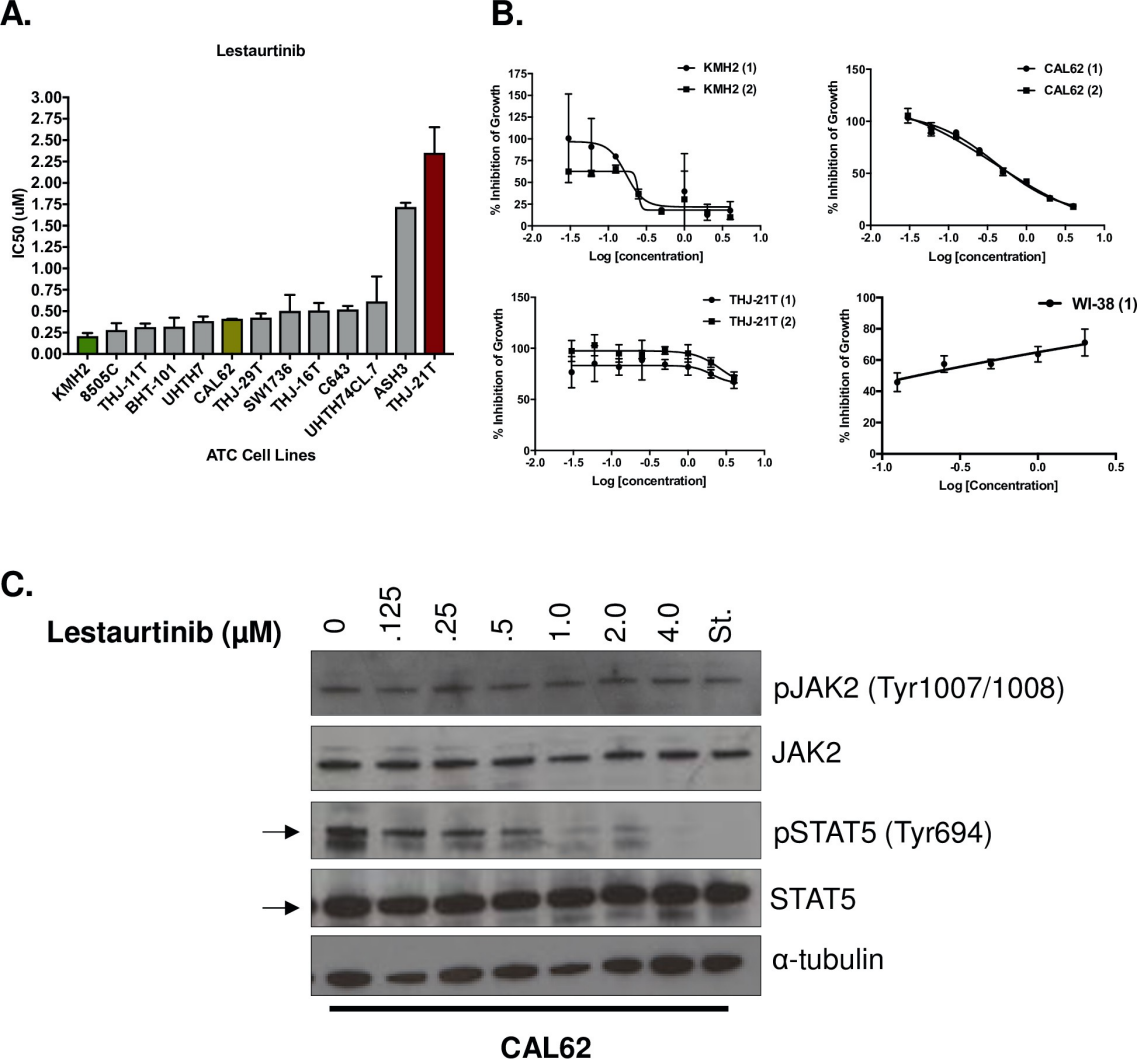
Lestaurtinib is identified as a potent agent across a panel of 13 ATC cell lines. **(A)** Lestaurtinib was tested against 13 ATC cell lines, with 2 biological replicates per cell line. Cells were treated with Lestaurtinib for 48 h and readings were completed after incubation with AlamarBlue. Mean IC_50_ values are shown ± standard deviation, stratified by lowest to highest mean values. **(B)** Dose-response curves demonstrating the effect of Lestaurtinib (μM) on cell growth following 72 h of treatment for KMH2, CAL62, THJ-21T (2 biological replicates) and WI-38 (1 biological replicate) cell lines. **(C)** Western blot of p-JAK2, JAK2, p-STAT5 and STAT5 showing protein expression of cells that were either untreated or treated with increasing concentrations (.125–4 μM) of Lestaurtinib, relative to the Staurosporine (St.) control.

We found Lestaurtinib, a JAK2 inhibitor, to be a highly potent agent across our cell line panel with 11 out 13 cell lines having nanomolar IC_50_ values. The cell line KMH2 was the most sensitive with a mean IC_50_ between both biological replicates of 0.21 ± 0.06 μM (**Fig 1A and 1B**). CAL62 was the sixth most sensitive, falling into the middle of our cell line panel, with a mean IC_50_ of 0.41 ± 0.001 μM (**Fig 1A and 1B**). THJ-21T was the most resistant cell line in our panel, with a mean IC_50_ of 2.35 ± 0.42 μM (**Fig 1A and 1B**). Lestaurtinib potency was also tested against the normal lung fibroblast cell line, WI-38, to measure how treatment with this drug would affect a normal cell line. Using a concentration range of 0.125–2 μM Lestaurtinib, we could not establish an IC_50_ for WI-38 because growth was not inhibited (**Fig 1B**).

We then sought to determine whether the measured sensitivity of ATC cell lines to Lestaurtinib could be explained by endogenous levels of JAK2 or its downstream target STAT5. Using Western blot analysis, we noted that neither the most sensitive lines (KMH2, 8505C and BHT-101) nor the most resistant lines (C643, ASH3 and THJ-21T), exhibited any correlation between the expression levels of endogenous JAK2 or STAT5 and sensitivity to Lestaurtinib treatment (**S1 Fig**). Given the high potency of Lestaurtinib and its’ potential as a therapeutic agent in our cell line panel, we selected both KMH2 and CAL62 cell lines for *in vitro* and *in vivo* validation studies.

To investigate whether Lestaurtinib was acting through the JAK2 pathway as predicted, we treated CAL62 with increasing concentrations of Lestaurtinib for 24 h. Treatment with Lestaurtinib did not result in a decrease of phospho-JAK2 (pJAK2) or endogenous JAK2; however, the downstream molecule STAT5 demonstrated a concentration-dependent decrease in phosphorylation starting at 0.125 uM (pSTAT5), with a complete disappearance of pSTAT5 expression at 4 μM (**Fig 1C**). For these reasons, we chose to use concentrations of 4 μM in our *in vivo* studies to determine the effects of inhibition of the JAK2 pathway with complete depletion of pSTAT5 expression.

### Lestaurtinib impaired colony formation and cell migration, *in vitro*

**Fig 2:**
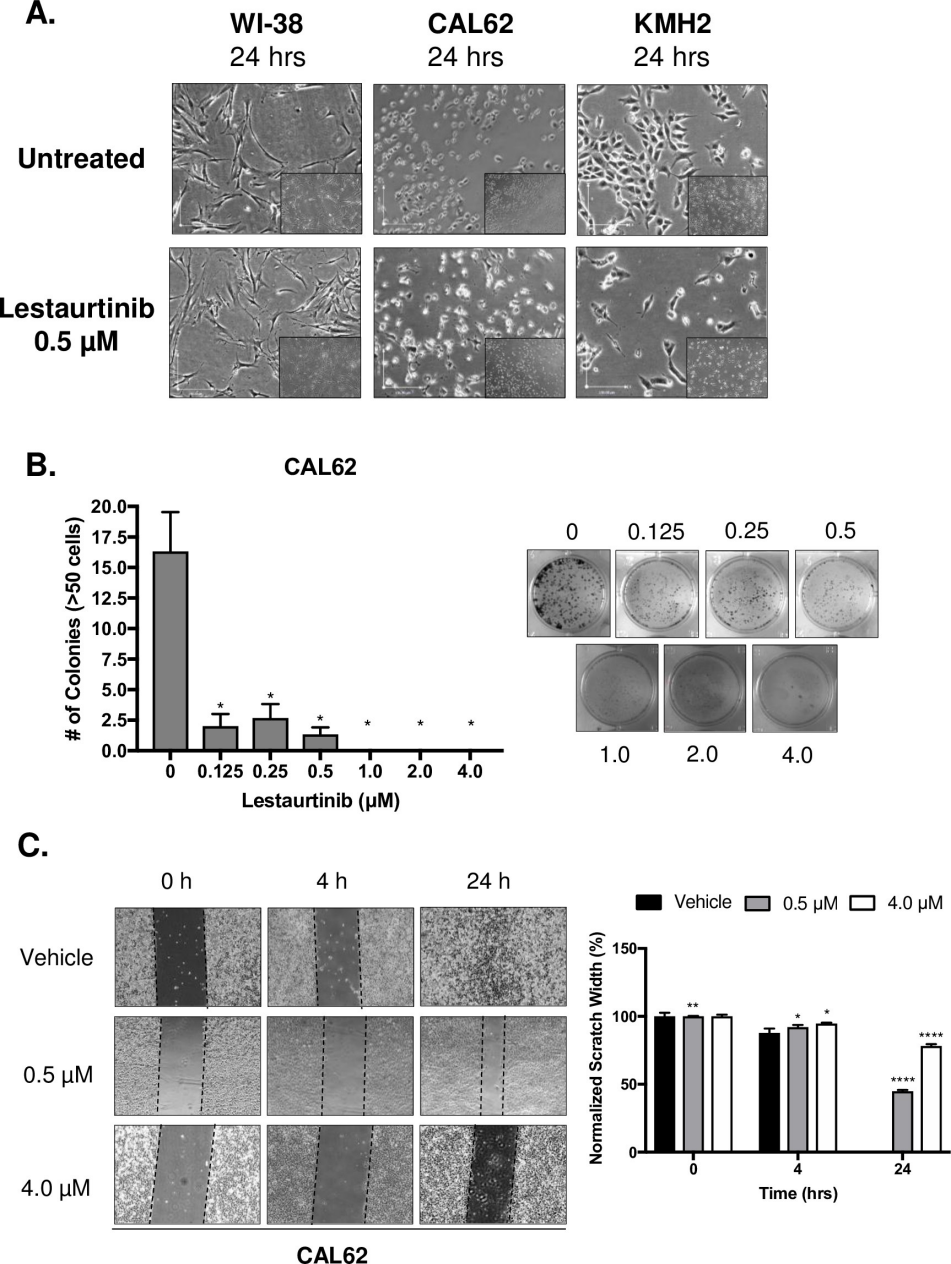
Treatment of ATC cell lines with Lestaurtinib resulted in an antiproliferative effect, *in vitro*. **(A)** Phase contrast images of WI-38, CAL62 and KMH2 cell lines with and without Lestaurtinib treatment (0.5 μM; 4X magnification). **(B)** Clonogenic assay of CAL62 both untreated and treated with increasing concentrations of Lestaurtinib (0.125–4 μM) and stained with 0.5% crystal violet. Colony formation was quantified by counting colonies from 3 representative fields, with colonies defined as being ≥50 cells. **(C)** Scratch-wound assay of CAL62 cells treated with 0.5 and 4.0 μM Lestaurtinib at 0, 4 and 24 h. Ten measurements were taken across the width of the scratch and averaged (3 replicates per cell line) per time point. * represents *p* < 0.05, ** represents *p* < 0.01, *** represents *p* < 0.001, **** represents *p <* 0.0001, ns = not significant, Student’s unpaired, two-tail *t*-test.

Normal (WI-38) and ATC (CAL62 and KMH2) cells were treated Lestaurtinib for a 24-hour period. Following 24 h of treatment, a concentration of 0.5 μM Lestaurtinib showed fewer cells with a rounder morphology in both CAL62 and KMH2 lines (**Fig 2A**). However, WI-38 cells treated with 0.5 μM were not inhibited (**Fig 2A**).

We next evaluated the effect of Lestaurtinib treatment on cells using a colony formation assay. Treatment of CAL62 with increasing concentrations of Lestaurtinib resulted in a significant inhibition of colony formation at all concentrations in the range of 0.125 to 4.0 μM when compared to the untreated control over a 7-day period (**Fig 2B**).

Previous studies identified a role for the JAK2-STAT pathway in cell migration[18–20], thus if Lestaurtinib acted on the JAK2-STAT pathway then we sought to determine how Lestaurtinib treatment would impact migration of CAL62 cells using a scratch-wound assay. At both 4- and 24 h, there was a significant difference in normalized scratch width percentage (%), where drug-treated scratches (0.5 and 4.0 μM) remained relatively open compared to the control (**Fig 2C**).

### Lestaurtinib treatment induced an antiproliferative effect with knockout of JAK2 in ATC cell lines

**Fig 3:**
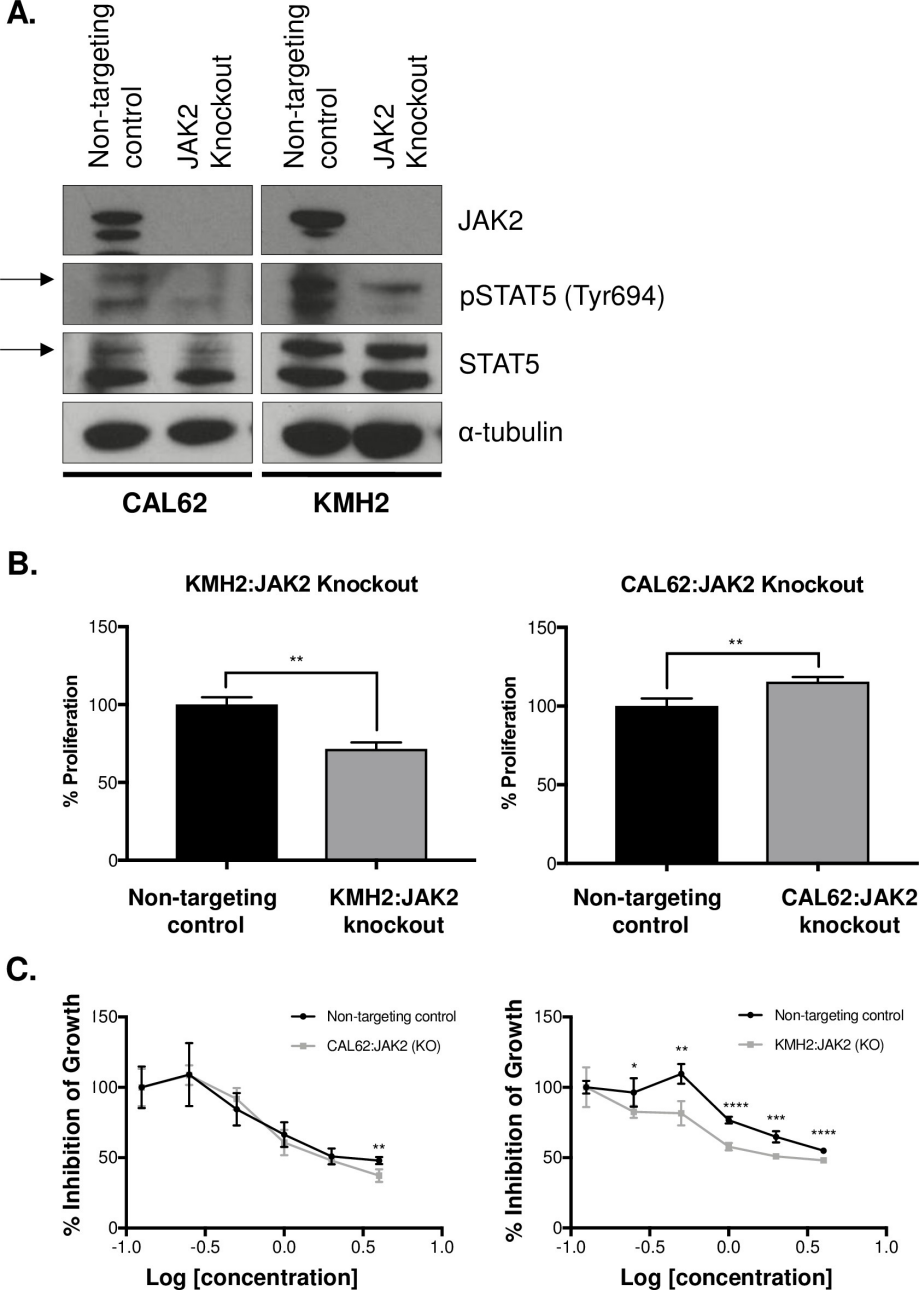
Treatment with Lestaurtinib resulted in an antiproliferative effect on JAK2 knockout cell lines. **(A)** Western blot showing loss of JAK2 expression following JAK2 knockout in CAL62 and KMH2 cell lines and compared to a non-targeting control. **(B)** Cellular proliferation of JAK2 knockout cell lines (CAL62 and KMH2) normalized to the non-targeting control and measured at 72 h. **(C)** Dose-response curves of CAL62 and KMH2 JAK2 knockout cells following 72 h of treatment with Lestaurtinib over a 6-point dose range (3 replicates per line). * represents *p* < 0.05, ** represents *p* < 0.01, *** represents *p* < 0.001, **** represents *p <* 0.0001, ns = not significant, Student’s unpaired, two-tail *t*-test.

We used CRISPR/Cas9 technology to knockout JAK2 in CAL62 and KMH2 cells, which would be expected to make the cells resistant to drug treatment. Complete knockout (KO) of JAK2 protein expression was achieved in both cell lines (**Fig 3A**). Furthermore, we noted that there was a decrease in STAT5 phosphorylation following JAK2 knockout, recapitulating one of the effects of Lestaurtinib treatment (**Fig 3A**). We then looked at cellular proliferation and found that the JAK2-edited CAL62 line exhibited a significant increase in cellular proliferation after 72 h compared to the non-targeting CRISPR control (*p =* 0.0090)(**Fig 3B**), while the KMH2 line demonstrated a significant decrease in cellular proliferation at 72 h compared to the non-targeting CRISPR control (*p =* 0.0014)(**Fig 3B**). Both JAK2 knockout cell lines were treated with Lestaurtinib over a 6-point concentration range to determine whether Lestaurtinib would impact cellular proliferation in the absence of JAK2. We observed a significant decrease in cellular proliferation of CAL62:JAK2 (KO) at the highest concentration (*p* = 0.0060) (**Fig 3C**). Furthermore, we observed a significant decrease in cellular proliferation of KMH2:JAK2 KO at every concentration of Lestaurtinib except the lowest (**Fig 3C**), suggesting that the antiproliferative effect of Lestaurtinib could not be explained solely through a JAK2 mechanism.

### Lestaurtinib treatment did not induce apoptosis, but induced cell cycle arrest in the G2/M phase

**Fig 4:**
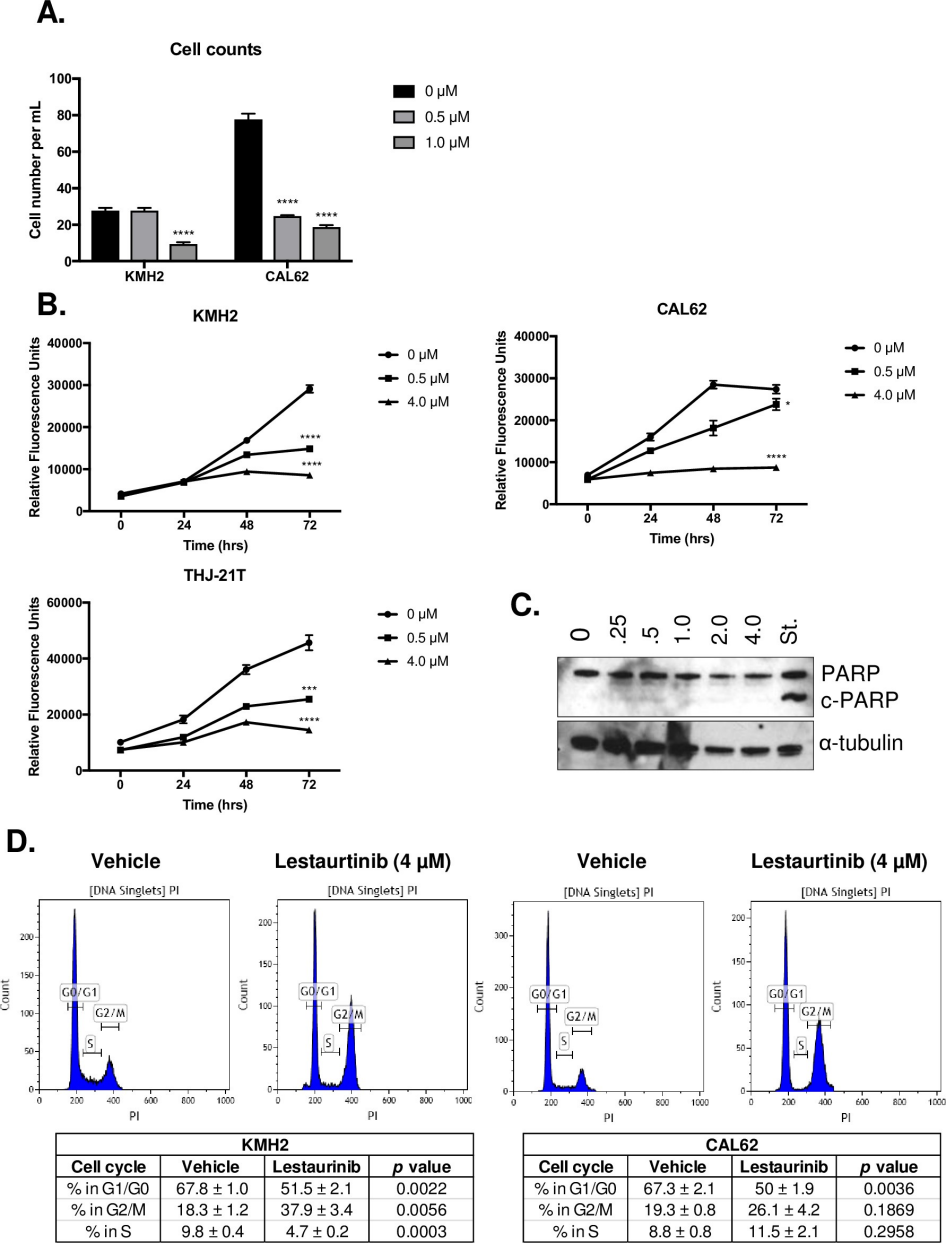
Lestaurtinib exhibited a cytostatic effect on ATC cells, *in vitro*. **(A)** Effect of Lestaurtinib (0.5 μM and 1.0 μM) on cell number following 24 h of treatment (3 replicates per line) for KMH2 and CAL62 cell lines. **(B)** Growth curves for KMH2, CAL62 and THJ-21T cell lines following treatment with Lestaurtinib at 0, 24, 48 and 72 h, with 3 technical replicates per timepoint. Statistical analysis is shown for the 72 h timepoint when compared to the untreated control for the same timepoint. **(C)** Western blot showing expression of cleaved PARP (c-PARP) following 24 h of Lestaurtinib doses (0.25–4.0 μM) relative to the apoptosis-inducing Staurosporine (St.) control. **(D)** CAL2 and KMH2 cells were treated with Lestaurtinib (4.0 μM) for 24 h (3 replicates per line) prior to BrdU incorporation and labeling of cells with propidium iodide. A minimum of 10,000 events were counted per test. Proportions of cells within each phase of the cell cycle is shown ± standard error. * represents *p* < 0.05, ** represents *p* < 0.01, *** represents *p* < 0.001, **** represents *p <* 0.0001, ns = not significant, Student’s unpaired, two-tail *t*-test.

We utilized a live-dead assay of CAL62 and KMH2 cells to determine the effect of Lestaurtinib on cell health. The number of cells significantly decreased when treated with 1 μM of Lestaurtinib when compared to the untreated control (*p* < 0.0001) after 24 h in KMH2 (**Fig 4A**). Furthermore, we noted that there was a significant decrease in cell number with both 0.5 (*p* < 0.0001) and 1.0 μM (*p* < 0.0001) after 24 h in CAL62 (**Fig 4A**). There was significant decrease in cell growth of Lestaurtinib treated KMH2, CAL62 and THJ-21T cell lines at the 72 h for both 0.5 and 4.0 μM treatments (**Fig 4B**). Western blot analysis was carried out to determine whether apoptosis was being induced following Lestaurtinib treatment. At all concentrations, cleaved PARP (c-PARP) was not detectable, while its presence was detected in cells treated with Staurosporine (St.) as a positive control (**Fig 4C**).

As the antiproliferative effects of Lestaurtinib did not appear to result in cell death, we next evaluated whether Lestaurtinib induced a cytostatic effect on ATC cell lines. CAL62 and KMH2 cells were treated with a concentration of 4 μM Lestaurtinib for 24 h and this resulted in a significant decrease in the number of cells in the G1/G0 phase (*p =* 0.0036) in CAL62 (**Fig 4D**). An increased number of CAL62 cells in the G2/M phase were also observed; however, this change was not significant (*p =* 0.1869) when compared to the vehicle-treated cells (**Fig 4D**). Lestaurtinib-treated KMH2 cells showed a significant increase in the number of cells in the G2/M phase (*p <* 0.0056), and significant decreases in the number of cells in the G1/G0 (*p <* 0.0022) and S (*p =* 0.0003) phases (**Fig 4D**), suggesting a decrease in the proportion of proliferating cells.

### Lestaurtinib restricted tumor growth and exhibited antiangiogenic activity in an *in vivo* chick CAM model

**Fig 5:**
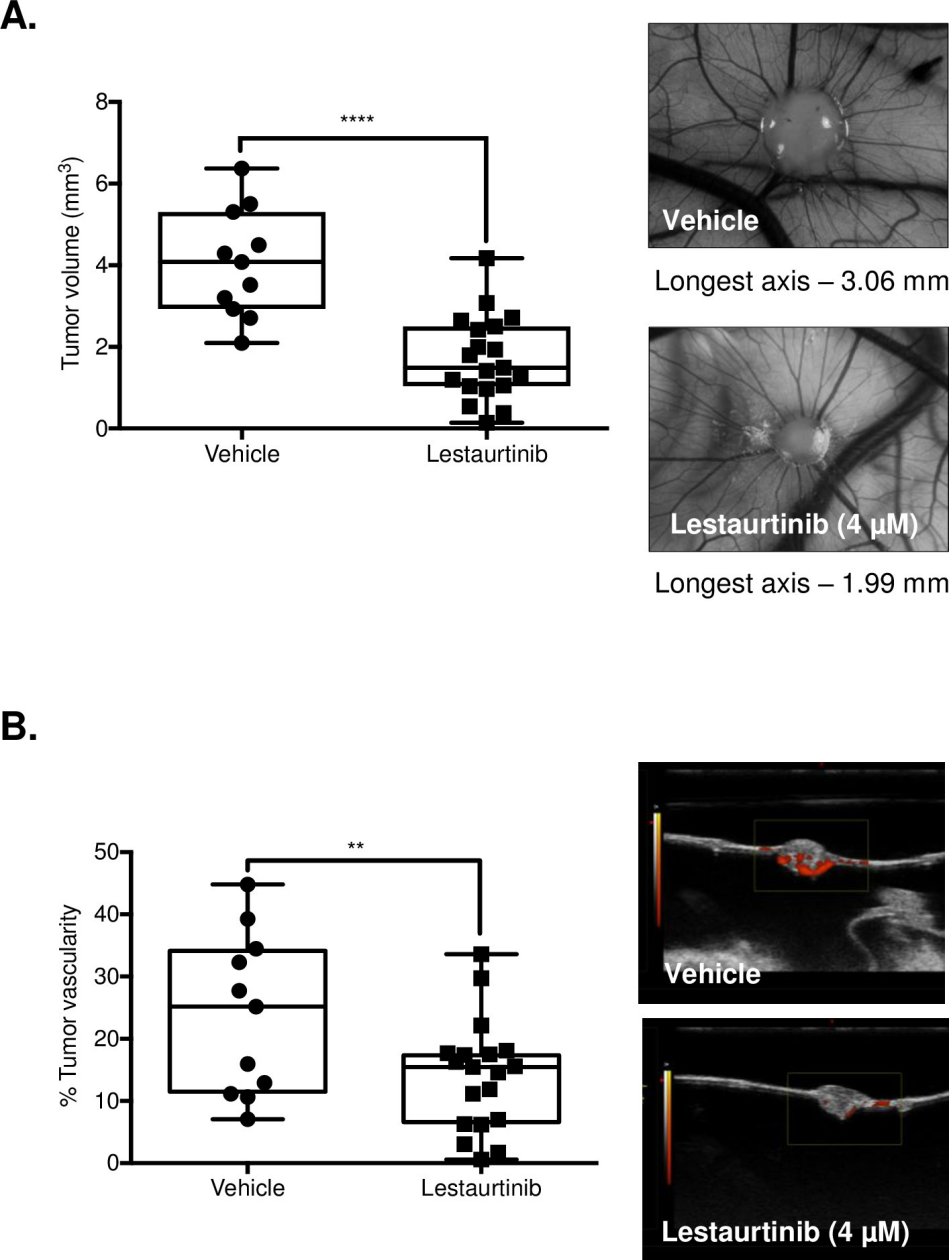
Lestaurtinib exhibits antiproliferative and antiangiogenic activity in the *in vivo* chick CAM membrane model. **(A)** A total of 1 x 10^6^ cells per embryo were administered over top (onplant) of the chick embryo membrane, 9 days post-fertilization. Two days post-onplant, embryos were either treated with the vehicle alone or Lestaurtinib (4 μM), once daily for 5 days and sacrificed. Mean endpoint tumor volume was measured using ultrasound imaging in the vehicle-treated (n = 11) and Lestaurtinib-treated (n = 19) embryos. **(B)** Power Doppler ultrasound imaging measured the intergroup difference in fractional vascular volume at endpoint to generate mean percent vascularity between groups. * represents *p* < 0.05, ** represents *p* < 0.01, *** represents *p* < 0.001, **** represents *p <* 0.0001, ns = not significant, Student’s unpaired, two-tail *t*-test.

The chick CAM model was used to examine the anti-tumor effects of Lestaurtinib in an *in vivo* model. CAL62 cells were used to establish xenograft tumors in CAM models. These were treated with either PBS (vehicle) or Lestaurtinib (4 μM) once a day for 5 days, with drug treatment beginning 2 days post-onplant. Mean endpoint tumor volume of vehicle-treated embryos (n = 11) was 4.05 ± 1.31 mm^3^, while that of the Lestaurtinib-treated embryos (n = 19) was 1.7 ± 1.02 mm^3^ (*p <* 0.0001; **Fig 5A**). Power Doppler ultrasound imaging was used to determine the inter-group difference in fractional vascular volume at endpoint. Using this modality, the mean percent vascularity decreased from 23.76 ± 12.95 to 13.99 ± 8.80% with Lestaurtinib treatment (*p =* 0.0202; **Fig 5B**). Together this data suggests that Lestaurtinib may act as an antiproliferative agent in ATC cell line models and its’ potential as an antiangiogenic agent warrants further investigation.

## Discussion

The aggressive and invasive nature of ATC, paired with the frequency of distant metastases seen in patients makes this disease very difficult to treat resulting in dismal patient outcomes[17]. Further, the rarity and rapid progression of this disease are considerable barriers for clinical trials. Thus, preclinical drug development is a crucial step to identify new targeted agents and the use of cultured cell lines for this purpose remains an important aspect of drug discovery due to their ease of manipulation and ability to test both *in vitro* and *in vivo*[7,21].

We identified the tyrosine kinase inhibitor Lestaurtinib as an inhibitor of cellular growth in ATC cells, with nanomolar IC_50_’s across the majority of the 13 ATC cell lines tested. Lestaurtinib has been previously used in several clinical trials for hematogenous malignancies, but it has not been explored for potential utility against solid malignancies such as thyroid cancer[22–25]. Furthermore, it has been studied extensively both *in vitro* and *in vivo*, and demonstrates a highly positive safety record and is orally bioavailable [23,24].

Lestaurtinib has been reported to be a multi-kinase inhibitor, specifically of the JAK2 signaling pathway[24]; however, many kinase inhibitors have off target effects[26,27], making their true anti-cancer mechanism unclear. In this study, we have reported the effects of Lestaurtinib *in vitro* and *in vivo*. Although we found evidence that Lestaurtinib did inhibit JAK2 pathway activation through a concentration-dependent decrease in downstream STAT5 phosphorylation, we also found that cells with JAK2 knockout demonstrated a similar or increased sensitivity to Lestaurtinib, indicating that the antiproliferative effects of Lestaurtinib may be due to off-target effects via the inhibition of other targets in addition to JAK2. Furthermore, we noted that the antiproliferative effects of Lestaurtinib were not mediated through apoptosis, but through cell cycle arrest with the accumulation of drug-treated cells in the G2/M phase of the cell cycle. Further studies are needed to uncover the true mechanism of action of this drug in addition to JAK2.

Xenografts were developed using the chick CAM model to understand the effects of drug treatment on tumor volume and vascularity using the CAL62 cell line [28–30]. Our *in vivo* studies demonstrated that treatment with Lestaurtinib resulted in a significant reduction in tumor volume. Furthermore, when we controlled for tumor volume, there was a significant decrease in the percent tumor vascularity when compared to the vehicle control, demonstrating a potential antiangiogenic effect that warrants further investigation. Together this data suggests that while Lestaurtinib demonstrates antiproliferative effects *in vitro*, there is potential for antiangiogenic effects which contribute to its overall antitumor activity.

In summary, our *in vitro* and *in vivo* data suggest that Lestaurtinib may induce its effects through the inhibition of JAK2 in addition to other off-target effects. Administration of Lestaurtinib demonstrated an antiangiogenic effect on ATC tumors, *in vivo*, with a relatively high drug dose used as a proof of principle, suggesting that this mechanism of action may be responsible for the antiproliferative effects observed in our study. Overall, the data in this study suggests that Lestaurtinib is an effective and valid therapeutic agent in the study of ATC, however, this drug warrants further investigation to understand the full potential as a targeted therapy in ATC.

## Supporting information

S1 Table Short tandem repeat profiling of 13 ATC cell lines. Cell lines were genotyped with 16 selected markers.
(XLSX)

S1 Fig Western blot analysis of ATC cell lines to determine endogenous levels of JAK2 and STAT5. Lysates from cell lines that appeared to be sensitive (KMH2, 8505C and BHT-101) and resistant (C643, ASH3 and THJ-21T) based on IC_50_ values were analyzed using Western blot analysis.
(TIF)
