# Supplementary figures and images for: Lestaurtinib is a potent inhibitor of anaplastic thyroid cancer cell line models
Source: PLoS One. 2018 Nov 12;13(11):e0207152. doi: 10.1371/journal.pone.0207152 (PMC6231667; doi:10.1371/journal.pone.0207152)

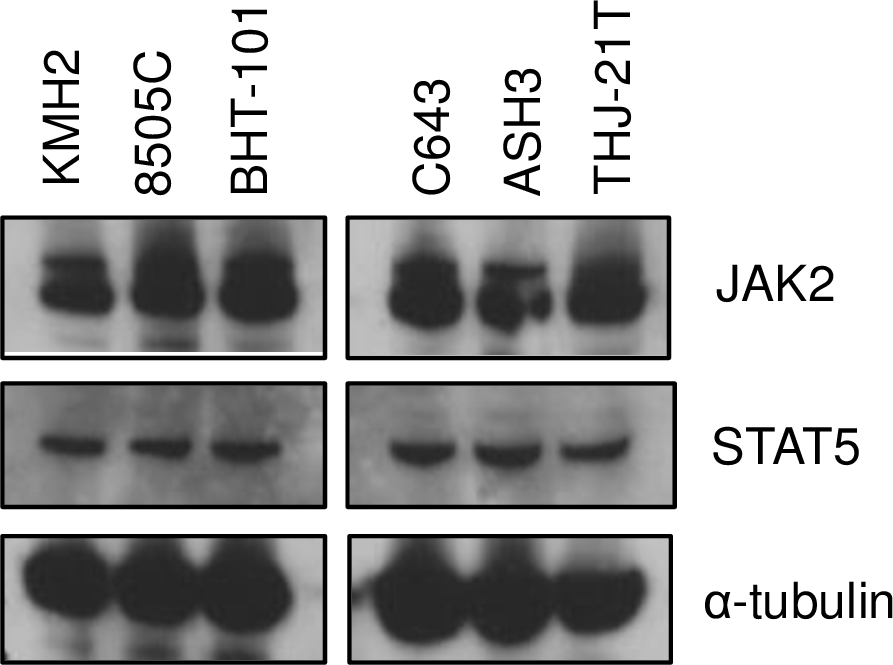

Supplement: S1 Fig — Lysates from cell lines that appeared to be sensitive (KMH2, 8505C and BHT-101) and resistant (C643, ASH3 and THJ-21T) based on IC50 values were analyzed using Western blot analysis. (TIF) [file pone.0207152.s002.tif]
